# Advancements in the Biotransformation and Biosynthesis of the Primary Active Flavonoids Derived from *Epimedium*

**DOI:** 10.3390/molecules28207173

**Published:** 2023-10-19

**Authors:** Xiaoling Zhang, Bingling Tang, Sijie Wen, Yitong Wang, Chengxue Pan, Lingbo Qu, Yulong Yin, Yongjun Wei

**Affiliations:** 1 School of Pharmaceutical Sciences, Zhengzhou University, Zhengzhou 450001, China; 2 Laboratory of Synthetic Biology, School of Pharmaceutical Sciences, Zhengzhou University, Zhengzhou 450001, China; 3 Key Laboratory of Food Safety Quick Testing and Smart Supervision Technology for State Market Regulation, Zhengzhou 450003, China; 4 College of Chemistry, Zhengzhou University, Zhengzhou 450001, China; 5 Institute of Subtropical Agriculture, Chinese Academy of Sciences, Changsha 410081, China

**Keywords:** *Epimedium*, flavonoids, pharmacological activities, extraction methods, biotransformation, biosynthesis

## Abstract

*Epimedium* is a classical Chinese herbal medicine, which has been used extensively to treat various diseases, such as sexual dysfunction, osteoporosis, cancer, rheumatoid arthritis, and brain diseases. Flavonoids, such as icariin, baohuoside I, icaritin, and epimedin C, are the main active ingredients with diverse pharmacological activities. Currently, most *Epimedium* flavonoids are extracted from *Epimedium* plants, but this method cannot meet the increasing market demand. Biotransformation strategies promised huge potential for increasing the contents of high-value *Epimedium* flavonoids, which would promote the full use of the *Epimedium* herb. Complete biosynthesis of major *Epimedium* flavonoids by microbial cell factories would enable industrial-scale production of *Epimedium* flavonoids. This review summarizes the structures, pharmacological activities, and biosynthesis pathways in the *Epimedium* plant, as well as the extraction methods of major *Epimedium* flavonoids, and advancements in the biotransformation and complete microbial synthesis of *Epimedium* flavonoids, which would provide valuable insights for future studies on *Epimedium* herb usage and the production of *Epimedium* flavonoids.

## 1. Introduction

The *Epimedium* genus, belonging to the Berberidaceae family, contains 68 species worldwide, with 58 of them (85.3%) distributed in China [1]. China is the center of geographical distribution and varieties of *Epimedium*. Over 15 *Epimedium* species have a long history of use in Traditional Chinese Medicine (TCM) and are believed to have kidney-nourishing and *Yang*-reinforcing properties [2]. Tao Hongjing, a renowned medical scientist, learned from shepherds that male sheep consuming a certain plant experienced significantly increased penile erections and mating frequency. Tao believed that this plant could enhance “*Yang*” energy, and named it “Yin-Yang-Huo” in Chinese [3]. It was later discovered that this was an *Epimedium* plant.

*Epimedium* was first mentioned over 2000 years ago in the “Shen Nong Ben Cao Jing”. It was later listed as a medium-grade herb in the “Ben Cao Gang Mu” by Li Shizhen during the Ming Dynasty [4]. In the *Chinese Pharmacopoeia* (2020 edition), *Epimedii Folium* (EF) refers to the dried leaves of four *Epimedium* plants, namely *E. brevicornum* Maxim, *E. sagittatum* (Sieb. et Zucc.) Maxim, *E. pubescens* Maxim, and *E. koreanum* Nakai [5]. EF is a classical herbal medicine. Alone, or combined within diverse prescriptions, it has been used to treat various diseases, including sexual dysfunction [6,7], osteoporosis [8], cancer [9], rheumatoid arthritis [10], and brain diseases [11]. Additionally, EF has been used in functional food production, and is available in alcoholic health beverages, health tea, and medicated gruel and noodle diets [2,12]. Published reviews have primarily concentrated on the compositions and molecular structures of the active components of EF, as well as the pharmacological activities of EF flavonoids [3,8,9,10,11]. In this review, we aim to emphasize the recent advancements in the biotransformation and biosynthesis of EF’s active flavonoids.

## 2. The Pharmacological Activities of Major *Epimedium* Flavonoids

**Figure 1:**
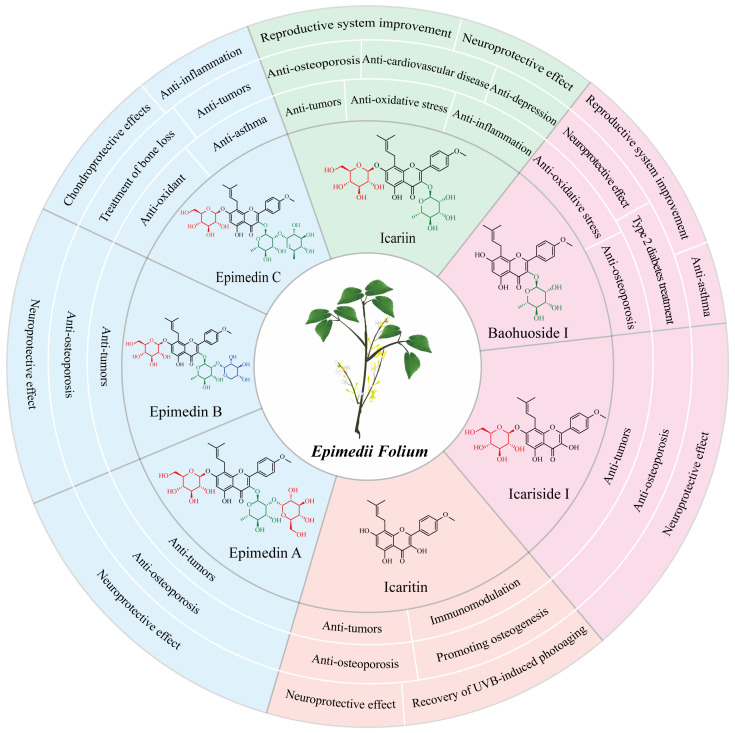
Chemical structures and pharmaceutical properties of the major flavonoids from *Epimedii Folium*.

More than 379 compounds have been detected in EF, including flavonoids, lignans, organic acids, terpenoids, dihydrophenanthrene derivatives, alkaloids, and other constituents [13]. Flavonoids, such as epimedin A, epimedin B, epimedin C, icariin, baohuoside I (also known as icariside II), icariside I, and icaritin have been recognized as major phytochemical and pharmacological active ingredients (Figure 1) [13,14]. These compounds differ in varying degrees of glycosylation at the C-3 and C-7 of icaritin [2,14]. There were great variations among the flavonoid contents in *Epimedium* from different species, collection and/or storage times and/or locations [15,16]. Icariin was the most abundant component in *E. brevicornum* Maxim and *E. koreanum* Nakai, followed by epimedin B, epimedin C, and epimedin A. However, epimedin C was the most abundant component in *E. sagittatum* (Sieb. et Zucc.) Maxim, *E. pubescens* Maxim, and *E. wushanense* T.S. Ying, followed by icariin, epimedin B, and epimedin A [15,16]. The average proportions of the total contents of epimedin A, B, C, and icariin to the 15 investigated flavonoid contents were 85.6%, 82.3%, 68.8%, 74.9%, and 69.8% in *E. brevicornum* Maxim, *E. koreanum* Nakai, *E. sagittatum* (Sieb. et Zucc.) Maxim, *E. pubescens* Maxim, and *E. wushanense* T.S. Ying, respectively [15,16]. In another study, epimedin A, B, C, and icariin accounted for over 52% of the total flavonoid contents in *E. brevicornum* Maxim [17]. In the *Chinese Pharmacopoeia* (2020 edition), the total amount of epimedin A, B, C and icariin was identified as the quality control indicator for the EF herb [5]. The major flavonoids in EF exhibit significant and diverse pharmacological activities (Figure 1).

### 2.1. Icariin and Its Pharmaceutical Effects

Icariin, the major bioactive component in EF (about 1%) [17], has been found to possess various pharmacological effects. These include improved reproductive system function, a neuroprotective effect, an anti-osteoporosis effect, protective effects from cardiovascular disease, an anti-inflammation effect, an anti-oxidative stress effect, an anti-depressive effect, and an anti-tumor effect [18,19,20,21]. In ancient China, EF was commonly used to treat sexual dysfunction [3]. Icariin can enhance erectile function in spontaneously hypertensive rats by reducing endothelial microparticle levels in the blood and inhibiting platelet activation [22]. In male mice, icariin can improve sexual function through the PI3K/AKT/eNOS/NO signaling pathway [23]. In the female reproductive system, icariin promotes estrogen biosynthesis in human ovarian granulosa-like KGN cells, and upregulates the expression of aromatase, which is responsible for the conversion of androgens to estrogens in vertebrates [24]. Additionally, icariin has exhibited protective effects in various nervous system disorders, including Alzheimer’s disease, Parkinson’s disease, and depressive disorder [20,25,26,27]. Moreover, icariin is regarded as a potential drug for osteoporosis treatment. Recent studies have demonstrated that icariin could prevent bone loss in ovariectomized rat models by modulating gut microbiota and regulating metabolite alterations [28] or by activating autophagy [29], as well as protect against iron overload-induced bone loss via suppressing oxidative stress [30].

### 2.2. Baohuoside I and Its Pharmaceutical Effects

Baohuoside I, although presents in low contents (<0.15%) in the raw material of EF compared to icariin, exhibits a wider range of pharmacological activities [31,32]. Baohuoside I has better bioavailability in vivo than icariin, as it is more easily absorbed by the capillaries of intestinal epithelial cells because of its lower polarity [33]. Cheng et al. found that 91.2% of icariin was converted to baohuoside I after oral administration in rats [34]. Similarly, human intestinal microflora metabolized most icariin to baohuoside I in a short time before absorption in the human intestine [35]. Baohuoside I has been proved to have a significant therapeutic effect on various diseases, such as sexual dysfunction, osteoporosis, and cancers [9,36,37,38]. For improving erectile dysfunction, baohuoside I could facilitate the differentiation of adipose-derived stem cells into Schwann cells and preserve the erectile function of bilateral cavernous nerve injury (BCNI) in rats [36,39]. The anti-osteoporotic activity of baohuoside I was suggested to be associated with its ability to induce bone marrow stromal cell differentiation into osteoblasts while inhibiting adipocyte formation, regulating immune functions, and providing antioxidant activity [40]. Baohuoside I could inhibit osteoclastogenesis and protect against ovariectomy-induced bone loss in mice, surpassing the effects of icariin [38]. Current studies have shown that baohuoside I exhibits promising anti-tumor effects on lung cancer cells [41], melanoma cells [42], breast cancer cells [43], prostate cancer cells [44], and osteosarcoma cells [45]. Furthermore, baohuoside I has shown its potential application in type 2 diabetes treatment [46], neuroprotection [47], and asthma inhibition [48].

### 2.3. Icaritin and Its Pharmaceutical Effects

Icaritin is a flavonoid aglycone in EF [14], which can be generated by hydrolytic reactions that remove the glycone parts of icariin, baohuoside I, and icariside I [49,50]. Icaritin possesses diverse pharmacological activities [51], including protection of neurons against amyloid-induced neurotoxicity [52], promotion of differentiation from embryonic stem cells into cardiomyocytes [53], anti-osteoporosis effects and osteogenesis promotion [54,55], immunomodulation [56,57], and recovery of UVB-induced photoaging of human keratinocytes [58]. Moreover, icaritin is considered as a promising candidate for the treatment of various cancers [59,60], including hepatocellular carcinoma [61,62,63], breast cancer [64], lung cancer [65], ovarian cancer [66], endometrial cancer [67], human oral squamous cell carcinoma [68], and multiple hematological malignancies [59,69,70,71]. In the treatment of hepatocellular carcinoma, icaritin can suppress cell growth and promote cell apoptosis via down-regulating alpha-fetoprotein gene expression in hepatocellular carcinoma [62] and inducing anti-tumor immune responses [61,63]. In 2022, an icaritin soft capsule was marketed as a small molecule immunomodulatory drug, providing a solution for patients with advanced hepatocellular carcinoma with poor prognosis, and significantly improving the life quality of patients with hepatocellular carcinoma [72,73].

### 2.4. Epimedin C and Its Pharmaceutical Effects

Epimedin C is a trioglycoside ingredient in EF, with the highest content among all flavonol glycosides in certain *Epimedium* species, such as *E. brevicornu* Maxim [74], *E. wushanense* T.S. Ying, and *E. sagittatum* Maxim [75]. Epimedin C is considered as the quality control standard for evaluating the quality of *E. wushanense* T.S. Ying in the *Chinese Pharmacopoeia* (2020 edition) [5]. The pharmacological activities of epimedin C mainly include treatment of bone loss, anti-oxidant effects, and anti-inflammation. Epimedin C has shown significant anti-inflammatory and chondroprotective effects by increasing the expression of extracellular matrix components in osteoarthritis chondrocytes [76]. Epimedin C could alleviate the suppressive impact of dexamethasone on the osteogenesis of larval zebrafish and MC3T3-E1 cells via triggering the PI3K/AKT/RUNX2 signaling pathway [77]. Notably, epimedin C has a stronger anti-osteoporosis effect than icariin at the same dose on dexamethasone-induced osteoporosis in a mouse model [78]. Furthermore, epimedin C has been found to protect against H_2_O_2_-induced peroxidation injury by enhancing the function of endothelial progenitor human umbilical vein endothelial cells, which plays an important role in repairing endothelial cell vascular injury [79]. In an ovalbumin-induced murine asthma model, epimedin C was demonstrated to dose-dependently decrease the protein levels of p52 and RelB, and the phosphorylation of ERK1/2, and p38 MAPK, which are pivotal in the development of Th9 cells and Treg cells, thereby inhibiting airway inflammation [80].

### 2.5. Other Flavonoids and Their Pharmaceutical Effects

Other flavonoids presented in EF include epimedin A and B, icariside I, and sagittatoside A, B, and C [2,13]. Their contents in EF are very low, and limited pharmacological research is available on them. However, similar to the major flavonoids described above, icariside I, epimedin A, and epimedin B also exhibit anti-osteoporosis, neuroprotective, and anti-cancer effects. Epimedin A has shown excellent efficacy against senile osteoporosis [8], and in vitro and in vivo experiments demonstrated that a complex epimedin A drug significantly enhances bone regeneration [81]. In addition, epimedin A could ameliorate 2,4-dinitrofluorobenzene (DNFB)-induced allergic contact dermatitis in mice, due to its ability to suppress the NF-κB/NLRP3 pathway, enhance the Nrf2 pathway, and modulate local inflammation [82]. Diao et al. provided evidence that epimedin B ameliorates osteoporosis in male mice via regulating PI3K-Akt, MAPK, and PPAR signaling pathways [83]. Additionally, epimedin B can exert a neuroprotective effect against Parkinson’s disease in an MPTP-induced mouse model [84]. Chen et al. suggested that icariside I performed tumor immunotherapy activity by blocking the kynurenine-AhR pathway and tumor immune escape [85]. Icariside I could significantly inhibit B16F10 melanoma growth in vivo through regulation of gut microbiota and host immunity [85]. Moreover, icariside I also effectively ameliorated estrogen deficiency-induced osteoporosis in an ovariectomy mouse model [86].

In addition to the various beneficial effects of EF flavonoids, it is important to note that EF can potentially cause drug-induced liver injury (Table 1). In clinical applications, there are increasing evidences indicate that Zhuangguguanjie pills and Xianlinggubao capsules have toxic effects, leading to liver injury in humans [87,88]. Both medicines contain EF as their major components, and are used to treat rheumatism, bone pain, arthritis, osteoporosis, and other diseases. Recently, animal studies have indicated that EF extracts can cause liver toxicity in mice and rats, with the severity of hepatotoxic effects increasing with higher dosages and prolonged exposure [89,90]. However, the exact compound(s) and the underlying mechanisms contributing to the observed liver toxicity remain unclear. Zhang et al. suggested that icariside I and sagittatoside A are the most relevant compounds related to the hepatotoxicity of EF extracts [91]. Epimedin C has been reported to have potential hepatotoxicity. Song et al. revealed that mRNA methylation might be associated with epimedin C-induced liver injury by the UPLC-MS/MS method [92]. When treated with the normal human liver cell line (HL-7702) and human hepatocellular carcinoma cell line (HepG2), 2″-O-Rhamnosyl icariside II, baohuoside I, and baohuoside II showed significant dose-toxic effects, and baohuoside I was more likely to be involved in the hepatotoxicity of EF [93]. Therefore, the hepatotoxicity of EF, like other TCMs, is probably due to the combined effects of multiple components. Further investigations are needed to fully understand the hepatotoxicity mechanism in order to avoid EF-induced liver injury.

## 3. The Biosynthetic Pathway of Prenylated Flavonoids in EF

**Figure 2:**
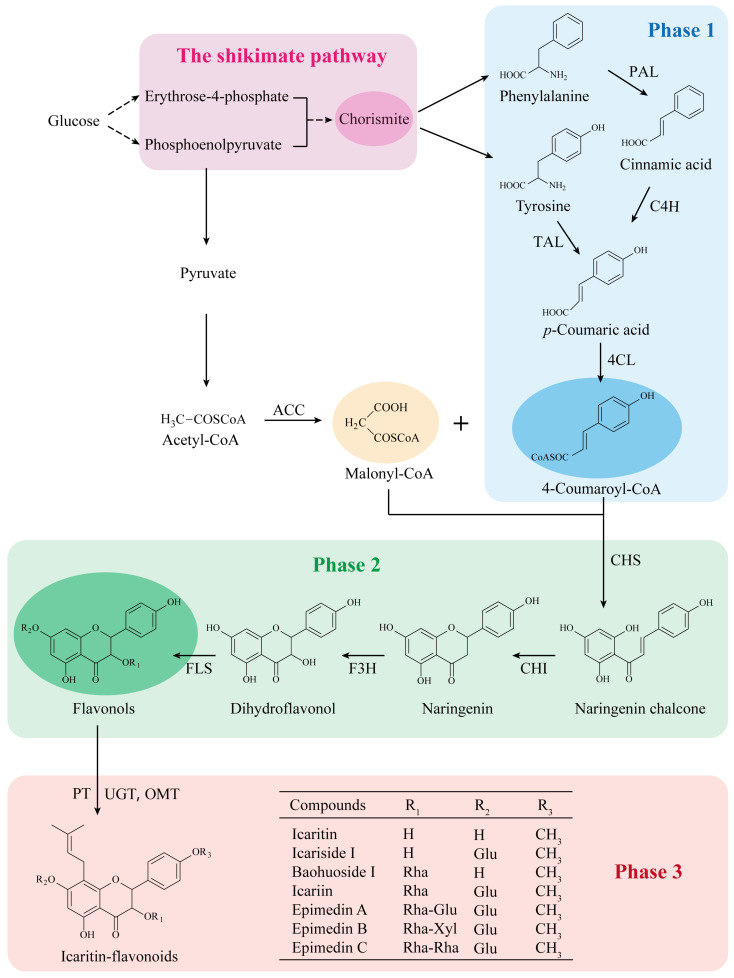
The biosynthetic pathway of prenylated flavonoids recovered from EF. Phase 1: the phenylpropanoid pathway; phase 2: the core pathway; phase 3: further enzymatic modification pathways. PAL: phenylalanine ammonia lyase, C4H: cinnamate-4-hydroxylase, TAL: tyrosine ammonia lyase, 4CL: 4-coumarate CoA ligase, ACC: acetyl-CoA carboxylase, CHS: chalcone synthase, CHI: chalcone isomerase, F3H: flavanone 3-hydroxylase, FLS: flavonol synthase, PT: prenyltransferase, UGT: UDP-glycosyltransferase, OMT: O-methyltransferase, Glu: Glucose, Rha: Rhamnose, Xyl: Xylose.

The biosynthetic pathway of *Epimedium* flavonoids has been explored in *E. sagittatum* Maxim and *E. pubescens* Maxim [95,96,97]. It can be divided into three phases (Figure 2): phase 1 involves the phenylpropanoid pathway, phase 2 refers to the core pathway, and phase 3 involves further enzymatic modification pathways [97]. The starting precursors for the phenylpropanoid pathway are phenylalanine and tyrosine, which are produced via the shikimate pathway [98]. In plant cells, glucose metabolism produces phosphoenolpyruvate (PEP) and erythrose-4-phosphate, and they are catalyzed by seven enzymes to convert to chorismite, which is the common precursor for the synthesis of phenylalanine and tyrosine [98,99].

In phase 1, phenylalanine is converted to cinnamic acid catalyzed by phenylalanine ammonia lyase (PAL); cinnamic acid is then catalyzed by cinnamate-4-hydroxylase (C4H) to produce *p*-coumaric acid, and tyrosine is converted to *p*-coumaric acid by tyrosine ammonia lyase (TAL) [100]. Then, 4-coumarate CoA ligase (4CL) converts *p*-coumaric acid to 4-coumaroyl-CoA. Another precursor, malonyl-CoA, is mainly derived from acetyl-CoA by acetyl-CoA carboxylase (ACC), and acetyl-CoA is often produced from the classic glucose metabolic pathway. In phase 2, one molecule of 4-coumaryl-CoA and three molecules of malonyl-CoA are condensed by chalcone synthase (CHS) to form naringenin chalcone (a C6-C3-C6 backbone unit for all flavonoids) [97,100]. Subsequently, naringenin chalcone is converted to naringenin by chalcone isomerase (CHI) to complete ring closing. Naringenin is transformed to dihydroflavonol by flavanone 3-hydroxylase (F3H). Flavonol synthase (FLS) further converts dihydroflavonol to flavonols. In phase 3, isopentenyl is added to flavonols by prenyltransferase (PT), and further modifications are carried out by various post-modification enzymes, such as UDP-glycosyltransferase (UGT) and O-methyltransferase (OMT), to produce a series of icaritin flavonoids, including epimedin A, epimedin B, epimedin C, icariin, baohuoside I, icariside I, and icaritin [96,97].

## 4. Extraction Methods of *Epimedium* Flavonoids

**Figure 3:**
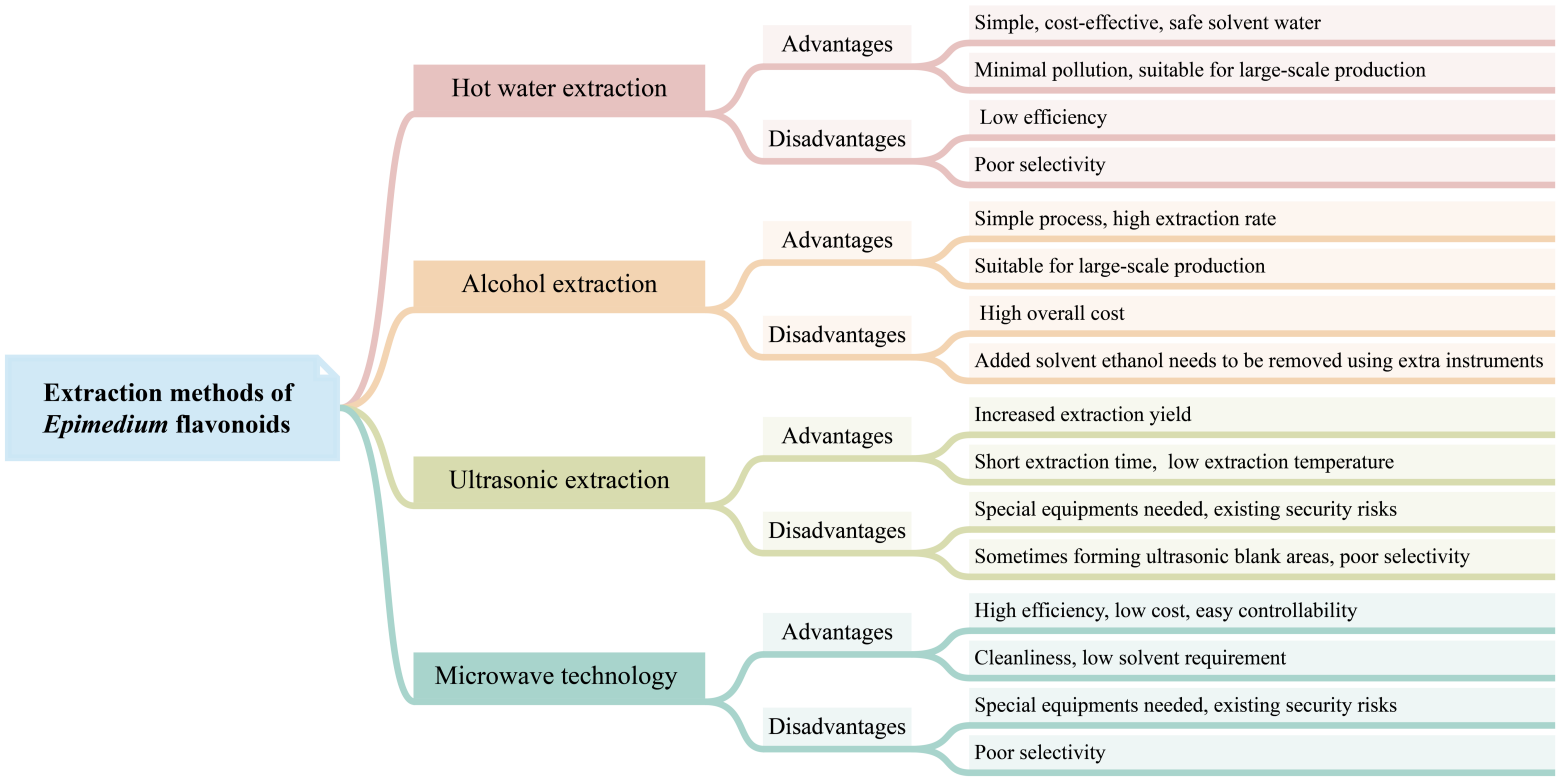
The advantages and disadvantages of different extraction methods of *Epimedium* flavonoids.

Currently, commercially available *Epimedium* flavonoids are extracted from *Epimedium* plants. Several techniques have been developed for isolating flavonoids from *Epimedium* (Figure 3), including hot water extraction, alcohol extraction, ultrasonic extraction, microwave-assisted extraction, and ultra-high-pressure extraction. Among these techniques, hot water extraction and alcohol extraction have been implemented in industrial production, while others are at the lab-scale stage.

### 4.1. Hot Water Extraction

Hot water extraction is a traditional method used for decocting Chinese herbs. In this method, the crushed herbs are immersed in water in a container for an appropriate amount of time, then heated and gently boiled for a certain period of time. The liquid is subsequently filtered, and the process of decoction is repeated 2–3 times. The decocted liquids from each iteration are mixed and concentrated to achieve the desired flavonoid concentration. Wang et al. optimized the hot water extraction procedure with an orthogonal test [101]. The results showed that the optimized extraction procedure was 2% Na_2_CO_3_, 15 times the water volume of the weight of dried material, with three 1.5 h extractions. The final extracting ratio of the total flavonoids was 97.92%. Other new technologies, such as microwave technology, have been used to enhance hot water extraction. Compared to the conventional hot water extraction method, microwave-assisted extraction offers higher extraction efficiency and is time-saving [102]. The hot water extraction process is simple and cost-effective, and utilizes water as a safe solvent. The whole process generates minimal pollution. Therefore, hot water extraction is suitable for the large-scale production of flavonoids. However, the efficiency of hot water extraction for flavonoid extraction is low, and it lacks selectivity in capturing specific flavonoids.

### 4.2. Alcohol Extraction

The alcohol extraction method is the most commonly used technique for extracting flavonoids, adopted by the *Chinese Pharmacopoeia* (2020 edition) [5]. In this method, ethanol is generally employed as the extraction solvent. The process of the alcohol extraction method is relatively simple, and well-suited for industrial applications. However, a large amount of ethanol is added to the extraction reactor, which subsequently needs to be removed using extra instruments. As a result, the overall cost of this method is higher compared to hot water extraction. Zhang et al. demonstrated that the extraction rate of icariin using the alcohol extraction method was significantly higher than that of the water extraction method [75]. The optimal extraction parameters were determined to be 50% ethanol, 1:10 solid–liquid ratio, 60 °C extraction temperature, 2 h extraction time, and two extraction cycles. In addition, an ultrasonic-assisted ethanol extraction procedure has shown to increase the extraction yield of epimedin A, epimedin B, epimedin C, and icariin from *Herba Epimedii*, when compared to the conventional ethanol boiling extraction method [75].

### 4.3. Other Extraction Methods

Ultrasonic extraction utilizes the effects of strong vibrations, cavitation, and thermal energy generated by ultrasound to extract the active components of plants into solvents. Ultrasonic extraction is regarded as a powerful tool for extracting flavonoids from plant biomass, offering several advantages, such as increased extraction yield, shorter extraction time, and lower extraction temperature [103]. Microwave technology utilizes the ability to generate heat within cells and vaporize water to break down the cell walls, allowing for better release of active ingredients in plant cells. The microwave technique presents numerous benefits, including high efficiency, low energy consumption, short processing time, low cost, cleanliness, easy controllability, and low solvent requirement [104]. Both ultrasonic extraction and the microwave technique are often used to assist common extraction methods, such as water extraction and alcohol extraction, to improve the efficiency of extracting *Epimedium* flavonoids [75,102,105,106]. Furthermore, ultra-high-pressure extraction has also been utilized for extracting flavonoids from *E. sagittatum*. Compared to heating extraction and ultrasonic-assisted extraction, ultra-high-pressure extraction presents distinctive advantages in superior extraction yield and a higher percentage of marker compounds [107].

## 5. Biotransformation and Biosynthesis of *Epimedium* Flavonoids

The natural resources of medicinal *Epimedium* species have experienced a significant decline over the past several decades, primarily due to over-harvesting and curtailment of habitats [108]. *Epimedium* is a low-growing perennial plant that is commonly propagated by rhizome division because of its low seed viability [109]. *Epimedium* needs specific growth conditions and thrives in acidic soil with shade and relatively high humidity [2]. The growth conditions directly affect the contents of the major components present in *Epimedium* plants, such as *Epimedium* flavonoids. The cultivation of large-scale *Epimedium* plants is challenging [110], as selecting high-quality and stable *Epimedium* species, advancing seedling propagation technology, and enhancing farmers’ planting income are difficult [111]. Thus, improving the use efficiency of the existing wild resources of *Epimedium* and exploring new alternative methods for producing *Epimedium* flavonoids to meet the huge market demand are necessary and of great interest.

### 5.1. Biotransformation of Epimedium Flavonoids by Enzymes

**Figure 4:**
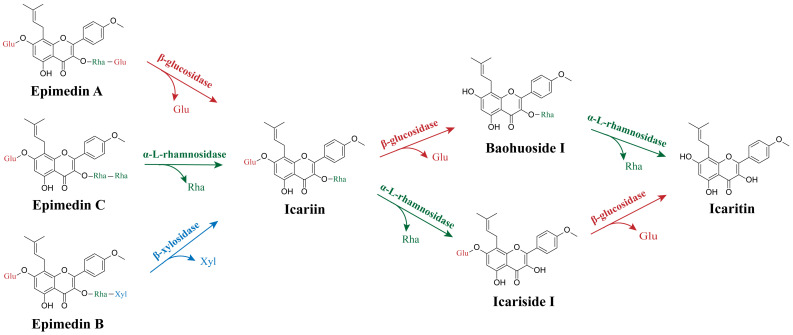
Schematic diagram of the transformation of high glycosylation flavonoids (epimedin A, epimedin B, and epimedin C) into low glycosylation flavonoids (icariin, baohuoside I, icariside I, and icaritin) by different enzymes. This conversion involves the enzymatic hydrolysis of the terminal sugar groups attached to the flavonoid molecules, resulting in the formation of the desired low glycosylation flavonoids.

*Epimedium* flavonoids mainly consist of epimedin A, epimedin B, epimedin C, icariin, baohuoside I, and icaritin. These compounds share a similar structure with the common aglycone skeleton icaritin, while differing in the type and number of lined sugar groups at the C-3 or C-7 positions [2,14]. Notably, icariin and baohuoside I are considered as the most effective components [18,33,112]. However, the contents of icariin and baohuoside I in *Epimedium* plants are extremely rare [17,32]. Therefore, converting other flavonoid components (epimedin A, epimedin B, and epimedin C) into icariin and baohuoside I through enzymatic hydrolysis of the terminal extra sugar groups, provides a feasible strategy to increase their contents in *Epimedium* extracts (Figure 4). Additionally, enzymatic hydrolysis shows enormous potential in the preparation of icariin and baohuoside I due to its notable selectivity, mild reaction conditions, high efficiency, and environmental friendliness [113,114].

The transformation of *Epimedium* flavonoids includes the hydrolysis of three types of sugar moieties: the glucose group, rhamnose group, and xylose group (Figure 1 and Figure 4). Three types of enzymes, namely glucosyl hydrolase, rhamnosyl hydrolase, and xylosyl hydrolase, are required to hydrolyze the corresponding sugar moieties (Table 2). High glycosylation flavonoids gradually hydrolyze sugar groups to form low glycosylation flavonoids (Figure 4). For example, epimedin C can be converted to icariin by removing its terminal rhamnose using α-l-rhamnosidase. To produce baohuoside I from icariin, β-glucosidase is employed to hydrolyze the glucose attached to the 7-O position of icariin. The aglycone icaritin can be generated by releasing the rhamnose moiety from the 3-O position of baohuoside I by α-l-rhamnosidase.

Previous studies have identified multiple enzymes capable of facilitating the conversion of *Epimedium* flavonoids (Table 2). Xie et al. discovered a novel thermostable GH78 family α-l-rhamnosidase (PodoRha) from *Paenibacillus odorifer*, which exhibited high specific activity in cleaving the outer α-1,2-rhamnopyranosyl moieties of epimedin C to produce icariin [114]. Another thermostable glucose-tolerant GH1 β-glucosidase (IagBgl1) derived from the hyperthermophile *Ignisphaera aggregans* was found to efficiently produce baohuoside I from icariin [32]. Based on these two enzymes, Xie et al. built a two-step conversion system consisting of PodoRha and IagBgl1 to transform epimedin C into baohuoside I with a conversion rate of 98% under optimized conditions [114]. A fungal α-l-rhamnosidase, AmRha, could hydrolyze the α-1,2-rhamnoside bond between two rhamnoses in epimedin C, achieving the production of icariin with a molar conversion rate of 92.3% in vitro [112]. When AmRha cooperated with β-glucosidase/β-xylosidase Dth3 [115], epimedin A, epimedin B, epimedin C, and icariin in the total *Epimedium* flavonoids could be converted to baohuoside I efficiently and simultaneously [112]. In addition, snailase, a kind of β-D-glucose hydrolase, was reported to contain more than 20 kinds of enzymes, including cellulase, pectinase, hemicellulose, and β-glucuronidase [116]. Snailase was applied to hydrolyze the total *Epimedium* flavonoids to the hydrolysate with enhanced anti-tumor activity [117].

**Table 2:** Three types of functional enzymes were used to transform *Epimedium* flavonoids.

Enzyme Type	Enzyme Name	Enzyme Source	Enzyme Characterization	Enzyme Functions	References
Rhamnosyl hydrolase	α-l-rhamnosidase (AmRha)	*Aspergillus* *mulundensis*	107.27 kDa, glycoside hydrolase (GH) 78 family; the optimal activity was achieved at 65 °C and pH 5.5, wide application temperature range, high level of enzyme catalytic ability and stability in the range of pH 5.0–7.5, acting on α-1,2-rhamnoside and α-1,6-rhamnoside bonds directly connected with glucose.	Catalyzes the bioconversion of epimedin C to icariin	[112]
	α-l-rhamnosidase	*Papiliotrema laurentii* ZJU-L07	100 kDa; the optimal activity was achieved at 55° C and pH 7.0, sensitive to temperature, stable at a pH range of 5.5–9.0 with an activity of over 80%, higher selectivity to cleave the α-1,2 glycosidic linkage between glucoside and rhamnoside and the α-1,2 glycosidic linkage between rhamnoside and rhamnoside.	Produces icariin from epimedin C	[118]
	α-l-rhamnosidase (PodoRha)	*Paenibacillus odorifer*	The molecular weight of the monomer was 100.12 kDa, the native recombinant PodoRha was a trimer, GH78 family; the optimal activity was achieved at 45 °C and pH 6.5, a broad range of activity within a pH range of 5.0–8.5, excellent thermostability at 40 °C and 35 °C, high specificity on α-1,2-glycoside in epimedin C.	Converts epimedin C into icariin	[114]
	α-l-rhamnosidase (DthRha)	*Dictyoglomus thermophilum* DSM3960	106.96 kDa, GH78 family; the optimal activity was achieved at 95 °C and pH 6.5, stable within the pH range of 4.5–7.5, residual activities exceeded 50% after incubation at 85 °C for 3 h and exceeded 90% after incubation at 75 °C for 3 h. Efficient hydrolyzation of the α-l-rhamnosidic bond of *Epimedium* flavonoids.	Converts epimedin C into icariin, converts icariin into icariside I, and converts baohuoside I into icaritin	[50]
	α-l-rhamnosidase (^syn^AnRhaE)	*Aspergillus nidulans*	95 kDa; the optimal activity was achieved at 55 °C and pH 4.5, stable in an acidulous pH range below 55 °C, high specificity on α-1,2 rhamnoside glycosidic bond in epimedin C.	Converts epimedin C into icariin	[119]
	α-l-rhamnosidase (SPRHA2)	*Novosphingobium* sp. GX9	120 kDa, GH106 family, when combined with PBGL, the optimal temperature for the reaction was 55 °C, and the highest activity was observed in 200 mM borate saline buffer at pH 8.5.	Catalyzes icariin into icariside I, converts baohuoside I into icaritin	[14]
	α-l-rhamnosidase (BtRha)	*Bacteroides thetaiotaomicron* VPI-5482	83.3 kDa, GH78 family; the optimal activity was achieved at 55 °C and pH 6.5, high selectivity to cleave the α-1,2 and α-1,6 glycosidic bond between rhamnoside and rhamnoside, rhamnoside and glycoside, respectively.	Transforms epimedin C to icariin	[120]
	α-l-rhamnosidase (Rhase-I)	*Talaromyces stollii* CLY-6	140 kDa, GH106 family; the optimal activity was achieved at 45 °C and pH 4.5, high thermal stability at a temperature lower than 50 °C and superior stability in an acidic environment (pH 2.0–5.0), be activated by Ca^2+^ and Mg^2+^, efficiently cleaving both the outer and inner rhamnosidic bonds of epimedin C.	Converts epimedin C into icariin, and converts icariin into icariside I	[121]
	α-l-rhamnosidase (AtRha)	*Aspergillus terreus* CCF3059	96.9 kDa, GH78 family; the optimal activity was achieved at 60 °C and pH 6.5, excellent thermal stability and pH stability, hydrolyzed icariin containing the α-1 rhamnoside linkage.	Hydrolyzes icariin to icariside I	[122]
Glucosyl hydrolase	β-glucosidase (Tpebgl3)	*Thermomotoga petrophila* DSM 13,995	81.24 kDa, GH3 family; the optimal activity was achieved at 90 °C and pH 5.0, the thermostability of the enzyme was improved by Ca^2+^, good stability at high temperatures and organic solvents.	Produces baohuoside I from icariin	[31,123]
	β-glucosidase (IagBgl1)	*Ignisphaera aggregans*	The molecular weight of the monomer was 56.36 kDa, the native recombinant IagBgl1 was a trimer, GH1 family; the optimal activity was achieved at 95 °C and pH 6.5, thermostable and glucose-tolerant, retained more than 70% after incubation at 90 °C for 4 h, high catalytic activity towards icariin.	Produces baohuoside I from icariin	[32]
	β-glucosidase (PBGL)	*Paenibacillus cookii* GX-4	84 kDa, GH3 family, when combined with SPRHA2, the optimal temperature for the reaction was 55 °C, and the highest activity was observed in 200 mM borate saline buffer at pH 8.5.	Converts icariin into baohuoside I, converts icariside I to icaritin	[14]
	β-1,3-glucanase (*Ct*Lam55)	*Chaetomium thermophilum*	82.7 kDa, GH55 family; the optimal activity was achieved at 60 °C and pH 5.0, thermostable at 50 °C, exo-β-1,3-glucanase activity.	Hydrolyzes icariin to baohuoside I	[113,124]
	β-glucosidase (Dth3)	*Dictyoglomus thermophilum* DSM3960	88.7 kDa, GH3 family; the optimal activity was achieved at 90 °C and pH 5.5, highly tolerant to glucose.	Converts epimedin A into baohuoside I, converts icariin into baohuoside I	[112,115]
	β-glucosidase	*Trichoderma viride*	60 kDa; the optimal activity was achieved at 41 °C and pH 4.0.	Prepares baohuoside I from icariin	[125]
	dextranase	*-*	The optimal activity was achieved at 40 °C and pH 5.4, sensitive to pH.	Hydrolyzes icariin to baohuoside I	[126]
	cellulase	*-*	The optimum conditions for the cellulase were 50 °C and pH 5.0.	Transforms icariin to baohuoside I	[127]
Xylosyl hydrolase	β-xylosidase (BbXyl)	*Bifidobacterium breve* K-110	70 kDa, GH43 family; the optimal activity was achieved at 45 °C and pH 5.5, the residual activity was more than 80% after being incubated at 45 °C for 4 h, showed over 70% of the maximum activity at a pH from 4.5 to 7.0, higher catalytic efficiency and selection specificity.	Converts epimedin B into icariin	[128]
	β-xylosidase (Dt-2286)	*Dictyoglomus turgidum*	85.1 kDa, GH3 family; the optimal activity was achieved at 98 °C and pH 5.0, excellent thermostable/haloduric/organic solvent-tolerance, a multifunctional enzyme with β-xylosidase, α-arabinofuranoside, α-arabinopyranoside and β-glucosidase activities.	Converts epimedin B into sagittatoside B, and converts sagittatoside B into icariside I	[129]
	β-xylosidase (Dth3)	*Dictyoglomus thermophilum* DSM3960	88.7 kDa, GH3 family; the optimal activity was achieved at 90 °C and pH 5.5, activity was not affected by xylose in high concentration.	Converts epimedin B into baohuoside I	[112,115]

-: Not determined.

### 5.2. Biotransformation of Epimedium Flavonoids by Whole-Cell Catalysis

**Figure 5:**
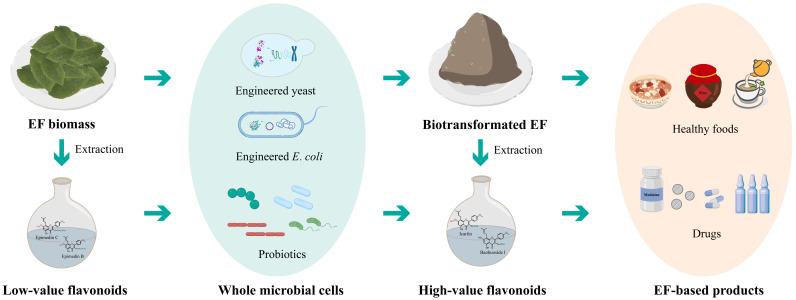
The work flow for biotransformation of *Epimedium* flavonoids by whole-cell catalysis, and their industrial applications.

Icariin, baohuoside I, and icaritin are the most effective components in *Epimedium* [31,118,119,122], and considered as high-value *Epimedium* flavonoids. Enzyme transformation is the preferred method for the preparation of icariin, baohuoside I, and icaritin, due to its high selectivity and catalytic efficiency, fewer by-products, mild reaction conditions, and convenient purification of the products [114,130]. However, enzymes, as a kind of biological macromolecule catalyst, have limitations in the large-scale use in industry, such as easily reduced activity and the high cost associated with enzyme expression and purification [131]. To address these challenges, enzymes are usually combined with carriers to improve their operational stability and reusability [131]. For example, Lu et al. successfully prepared an immobilized enzyme (4LP-Tpebgl3@Na-Y) by fusing the 4LP linker to thermostable β-glucosidase (Tpebgl3) on Na-Y zeolite. This immobilized enzyme exhibited enhanced tolerance of organic solvent and glucose, higher activity, and more reuse cycles, as compared to free enzymes during the production of baohuoside I from icariin [31]. Whole-cell catalysis is an alternative strategy that eliminates enzyme isolation and purification processes, thereby reducing costs (Figure 5). In this strategy, the cells provide a natural and protective environment for the enzymes [132]. Therefore, hydrolysis of flavonoid glycosides by whole-cell catalysis has the potential to be a low-cost strategy while retaining high efficiency.

Several studies have used whole microbial cells to transform *Epimedium* flavonoids. The α-l-rhamnosidase ^syn^AnRhaE from *A. nidulans* was expressed in *Escherichia coli*, and the recombinant *E. coli* could efficiently transform epimedin C to icariin [119]. Another α-l-rhamnosidase SPRHA2 and β-glucosidase PBGL were expressed in *E. coli*, respectively. The mixture of SPRHA2 cells and PBGL cells was used to transform icariin into icaritin, and the yield rate was 95.23%. Moreover, crude icariin extracts were also efficiently hydrolyzed by the recombinant *E. coli* strain that co-expressed SPRHA2 and PBGL [14]. Additionally, the recombinant *E. coli* strain transformed with α-l-rhamnosidase BtRha and β-xylosidase BbXyl could convert the total flavonoids of *Epimedium* into icariin [128]. Furthermore, the engineered *Komagataella phaffii* GS115 strain expressed with α-l-rhamnosidase AmRha could transform epimedin C to icariin efficiently. This strain also largely removed the feedback inhibition of l-rhamnose and enabled a five-fold increase in the concentration of raw *Epimedium* flavonoids [112]. Whole-cell catalysis, as an intermediate approach between fermentation and in vitro enzyme catalysis, offers several advantages over the use of purified or immobilized enzymes. These include significantly reduced catalyst costs, increased stability due to residual cell wall compounds, and no need for external cofactor addition [133]. Overall, these findings highlight the potential of whole-cell catalysis as a promising approach for future industrial production of high-value *Epimedium* flavonoids.

### 5.3. Complete Biosynthesis of Epimedium Flavonoids

Biotransformation processes have demonstrated the ability to convert low-value flavonoids into high-value flavonoids. However, the production of natural products from plant biomass requires large amounts of arable land and labor-intensive cultivation. Metabolic engineering and synthetic biology methods have emerged as alternative, simple, and eco-friendly strategies for the production of high-value natural herb products [134,135]. Currently, microbial cell factories have been built to produce diverse natural products, such as artemisinic acid [136], opioids [137], and glycyrrhetinic acid [135].

The biosynthetic pathways of specific *Epimedium* flavonoids have been elucidated, and successful production of icaritin has been achieved. Wang et al. utilized a metabolic engineering strategy to build yeast strains by introducing 11 heterologous genes and modifying 12 native yeast genes. After relocating the methyltransferase GmOMT2 into yeast mitochondria or co-culturing the engineered yeast with an *E. coli* strain expressing GmOMT2, they realized the biosynthesis of icaritin from glucose, and the yields of icaritin were 7.2 and 19.7 mg/L [110].

**Figure 6:**
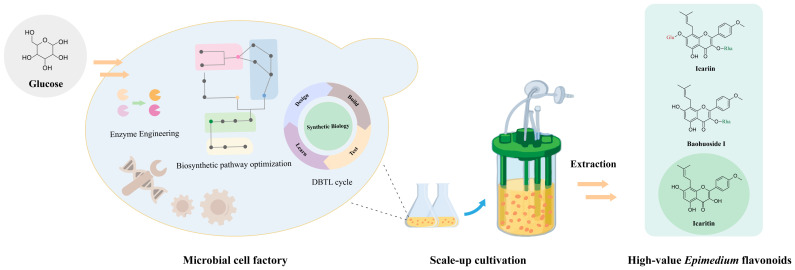
Complete biosynthesis of *Epimedium* flavonoids in a microbial cell factory.

The biosynthesis of other *Epimedium* flavonoids, such as monoglycoside flavonoids of baohuoside I and icariside I, diglycoside flavonoid of icariin, and trioglycoside flavonoids of epimedin A, B, and C, requires specific glycosyltransferase, rhamnosyltransferase, xylosyltransferase, and other modifying enzymes [97]. Some of these enzymes are not well characterized, and their compatibility with eukaryotic chasses and the sequential reactions involved in *Epimedium* flavonoid biosynthesis have not yet been determined. Despite these challenges, the application of metabolic engineering and synthetic biology holds great potential for advancing the biosynthesis of *Epimedium* flavonoids (Figure 6). Future research efforts focused on characterizing enzymes, improving compatibility with yeasts and other microbial hosts, and optimizing biosynthetic flavonoid pathways will contribute to the de novo production of *Epimedium* flavonoids in microbial cell factories.

## 6. Perspective

EF, an important TCM, has been safely used for more than 2000 years to treat various diseases, such as sexual dysfunction, osteoporosis, rheumatoid arthritis, cardiovascular disease, nervous system diseases, and tumors [138,139,140,141,142,143]. Modern evidences have confirmed that the major active pharmacological ingredients of EF, including epimedin A, epimedin B, epimedin C, icariin, baohuoside I, and icaritin, have corresponding pharmacological activities.

To enhance the production of bioactive high-value *Epimedium* flavonoids, multiple methods have been developed, including biotransformation by enzymes or whole cells [113,115], chemical synthesis [144], and biological synthesis by microbial cell factories [107]. Among these methods, whole-cell catalysis is regarded as a low-cost, stable, highly efficient, and environmentally friendly strategy. Actually, the treatment of Chinese herbs with microorganisms is a traditional technique; TCM fermentation was recorded thousands of years ago in China [145]. Fermenting Chinese herbs with microorganisms, especially probiotics, could promote the release of effective ingredients, reduce toxicities, generate new bioactive substances, enhance bioavailability, and improve pharmacological activities [146]. Fermenting EF with *Lactobacillus plantarum* has shown to increase the relative contents of flavonoid aglycones, monoglycosides, and phenolic acids, while the relative contents of multilevel and secondary flavonoid glycosides decreased, and the antioxidation of fermentation products was significantly increased [147].

Currently, synthetic biology is a powerful tool for the production of complex and high-value natural products using microbial cell factories [136]. However, the recovery of the key enzymes and building of the desirable engineered microorganisms remain challenging tasks. Omics technologies have been widely used to identify new functional genes, while metabolic engineering and metabolic flux analyses help to build complete and efficient natural-product synthetic pathways in microbial cell factories. These advanced technologies would greatly promote the efficient production of Chinese herb-derived bioactive ingredients in the near future, including *Epimedium* flavonoids.

## Figures and Tables

**Table 1:** The published mechanisms of the hepatotoxicity effects of *Epimedium* flavonoids.

*Epimedium* Flavonoids	Research Systems	Mechanisms	Reference
Alcohol extracts of *E. koreanum* Nakai and *E. wushanense* T.S. Ying	SD rats	Compared with the normal group, animal groups treated with EF extracts showed severer hepatotoxicity, which was positively correlated with the dose and course. Additionally, the females experienced more significant damage compared to the males.	[90]
Icariside I and sagittatoside A	HL-7702 and HepG2 cells	Icariside I could destroy the cell structure and cause oxidative stress. Sagittatoside A could cause oxidative stress and damage to mitochondria.	[91]
Epimedin C	Male Balb/c mice	Epigenetic modification changed in mouse liver after epimedin C treatment with a test dose, and the m^6^A and m^5^C may be associated with epimedin C-induced liver injury.	[92]
Baohuoside I	HL-7702 and HepG2 cells	The toxicity mechanism(s) of baohuoside I may be involved in increasing oxidative stress and inducing apoptosis.	[93]
*E. koreanum* Nakai ethanol extract	Male Sprague Dawley rats	The mechanism of hepatotoxicity of *E. koreanum* Nakai was probably related to the induction of ferroptosis in hepatocytes.	[94]

## Data Availability

Not applicable.
